# Bringing Older Adults to the Table: Opportunities for Consumer Engagement in Planning and Policy Making

**DOI:** 10.1093/geroni/igaa057.337

**Published:** 2020-12-16

**Authors:** Alice Prendergast, Kristi Fuller

**Affiliations:** Georgia State University, Atlanta, Georgia, United States

## Abstract

Efforts to include community voice in health policy and service planning are gaining recognition and support in the United States. Findings suggest community involvement can contribute to a better understanding of systems and factors that impact health, and, subsequently, more effective and sustainable policy and program design. Additionally, engagement can increase community buy-in, and community members can gain a greater awareness of services; increased confidence navigating systems; feelings of social connectedness; and capacity to advocate around issues through participation. Despite these findings, the extent to which community members are engaged in planning and decision-making varies considerably. Researchers from Georgia State University conducted a review of state plans on aging using the Person-Centered Outcomes Research Initiative (PCORI) Engagement Principles and the Health Research & Educational Trust’s Community and Patient Engagement Spectrum as frameworks to assess evidence of community engagement. The frameworks recognize engagement throughout the planning process, including design, data collection and interpretation, and dissemination. The review revealed that few planning processes described significant engagement, but rather met the minimal requirements established by federal policy. Federal guidance on community-informed planning practices is sparse, as are resources to support states in adopting these processes. To address this gap, the research team drew on the frameworks and other promising practices to design two community engagement projects, both in partnership with Georgia’s Division of Aging Services. Methods for participant engagement, data collection, interpretation and application of results, and lessons learned through both projects will be discussed, as well as potential implications.

